# Modelling of the breadth of expression from promoter architectures identifies pro-housekeeping transcription factors

**DOI:** 10.1371/journal.pone.0198961

**Published:** 2018-06-21

**Authors:** Lukasz Huminiecki

**Affiliations:** Instytut Genetyki i Hodowli Zwierząt Polskiej Akademii Nauk, Jastrzębiec, Magdalenka, Poland; Università degli Studi di Milano, ITALY

## Abstract

Understanding how regulatory elements control mammalian gene expression is a challenge of post-genomic era. We previously reported that size of proximal promoter architecture predicted the breadth of expression (fraction of tissues in which a gene is expressed). Herein, the contributions of individual transcription factors (TFs) were quantified. Several technologies of statistical modelling were utilized and compared: tree models, generalized linear models (GLMs, without and with regularization), Bayesian GLMs and random forest. Both linear and non-linear modelling strategies were explored. Encouragingly, different models led to similar statistical conclusions and biological interpretations. The majority of ENCODE TFs correlated positively with housekeeping expression, a minority correlated negatively. Thus, housekeeping expression can be understood as a cumulative effect of many types of TF binding sites. This is accompanied by the exclusion of fewer types of binding sites for TFs which are repressors, or support cell lineage commitment or temporarily inducible or spatially-restricted expression.

## Introduction

After integrating FANTOM5 (F5) and ENCODE datasets, Hurst *et al*. [1] demonstrated that a simple metric of the architecture of the proximal promoter, namely its size (that is the cardinality of the set of interacting TFs), was strongly correlated with the breadth of expression (BoE). Statistical analyses, and the knowledge about the molecular mechanism of the activation of transcription [2], suggested that this correlation was causative. This is in contrast to associations with various metrics of gene expression described previously for chromatin features such as histone acetylation or histone H3K4/36/79 methylation [3], or the DNase I signal [4], or gene compactness/codon usage [5]. The latter associations may still be useful for prediction, but they most likely cannot explain the molecular mechanism of the initial activation of transcription (they are effects, not causes, although chromatin modifications may help in the propagation of the activated transcriptional state).

We need to start with a few definitions. BoE means the fraction of tissue- and cell-types in which a gene is expressed. It is arguably the most fundamental metric of gene expression in multicellular animals. BoE is of particular interest in vertebrates, which are characterized by complex development and body plans accompanied by the existence of hundreds of different cell and tissue types. Two categories of genes, namely housekeeping and tissue-specific, stand at the opposing ends of the spectrum of BoE. A housekeeping gene is expressed in all tissues and has a high BoE (equal to or approaching one). A tissue-specific gene is expressed in just a fraction of tissues and has a low BoE (below 0.33).

For the purpose of clarity, we also need to introduce ENCODE and F5 datasets. ENCODE stands for the Encyclopedia of DNA Elements; the ENCODE consortium focused on the annotation functional elements in the human genome and included ChIP-seq data for 161 TFs [6]. Project F5 was the fifth edition of the international consortium for the Functional Annotation of the Mammalian Genome [7]. The project produced a comprehensive catalogue of expression patterns, at a single-base resolution, in human and mouse tissues, which was used by Hurst *et al*., and re-used here, to calculate BoE. We underline that the strategy of integrative bioinformatics and data-mining allows one to merge ENCODE with F5 to answer biological questions that could not be tackled using either of the datasets alone.

Importantly, the findings reported by Hurst *et al*. were robust. The correlation between BoE and the size of promoter architecture held across different sample categories of the F5 catalogue: bulk tissues, primary cells, and cancer cell-lines. Crucially, the effect was also robust to the variation in how BoE and the proximal promoter were defined. Varied cutoffs and window sizes were used, all uniformly leading to the same conclusion: the more TFs a proximal promoter interacted with, the more likely it was to drive housekeeping expression.

What is meant by the architecture of the proximal promoter? The promoter architecture means the *set* of TFs which can bind the promoter [8]. (It is noted that alternative definitions of promoter architectures are possible and might be more useful for some purposes. In the definition of promoter architecture, one might include cell-specific and/or transient epigenetic modifications or protein-DNA interactions.) Multiple binding sites can be modelled if a *multiset* rather than a set is used to mathematically represent such promoter architectures. (Multiset is a generalization of the mathematical concept of set, where multiple occurrences of the element are modelled using multiplicities.) This can be advantageous as multiple TF sites of the same type are likely to have additive effects on gene expression. The size of an architecture means the cardinality of its set or multiset representation. This number expresses how many different TFs can bind the proximal promoter.

The above definition of the promoter architecture is known as *global* and *static* [8]. It implies the merging of ENCODE ChIP-seq peaks across different cell- and tissue-types. The resulting architecture is independent of developmental stage or environmental conditions. Metaphorically speaking, such promoter architectures are more alike a menu of TFs from which a promoter can “choose”, and less like a snapshot of TFs actually bound in any particular cell type. Global and static promoter architectures have proven a useful research tool [1, 9]. What is important is that with global and static promoter architectures, one can predict and explain expression patterns in cell- and tissue-types which are not matched with ENCODE’s, re-using and generalizing ENCODE datasets. This is exceptionally valuable as ENCODE datasets are not easily expanded or repeated.

The findings reported by Hurst *et al*. were not only robust, but also had a predictive value. Predictions could be made using a simple linear model, but a support vector machine—SVM [10], more precisely its *libsvm* implementation [11], had superior performance for predicting BoE. This suggested that the types of transcription factors (TFs) bound did matter, not just the total number. However, Hurst *et al*. did not determine which TFs matter most, what their precise quantitative contributions were, and whether any TFs tended to be excluded from the architectures of the promoters of housekeeping genes. (SVMs lack in interpretability. Support vectors are abstract measures that define a hyper-plane in much higher dimensions than we are used to perceiving. These vectors are not directly interpretable as correlation coefficients are.) The aim of the current study is to fill this knowledge gap, presenting a more quantitative picture of the regulation of BoE. For this purpose, we intentionally concentrate on explanatory rather than predictive modelling. However, we do verify our models by cross-validation as it is suggested in statistical literature [12].

The distinction between predictive and explanatory statistical modelling was underlined by Galit Shmueli in an influential review [13]. The ultimate goal of explanatory modelling is to infer causal relationships and to create new useful theoretical generalizations. (However, the descriptive modelling of associations between variables without assuming causality can be also useful in most cases.) Non-linear methods such as SVMs are ill-suited for this type of statistical modelling. Instead, one ought to use generalized linear models (GLMs), which are a robust generalization of multiple regression [14]. In the GLM models presented here, the coefficients of multiple regression should be interpreted as the effect of the given TF on BoE over and above all other TFs included in the model formula.

The reader is sure to be aware that there are two major approaches to statistical modelling: classical and Bayesian. In the classical approach, parameters are point estimates, usually calculated according to the method of maximum likelihood. The data are seen as a random variable. In the Bayesian approach, this situation is reversed. Parameters are modelled as random variables with assumed prior distributions that are updated given observed data according to the Bayes rule. By using both the classical approach and the Bayesian approach, we ensure that the findings reported here are robust in respect to the statistical methodology used.

**Fig 1:**
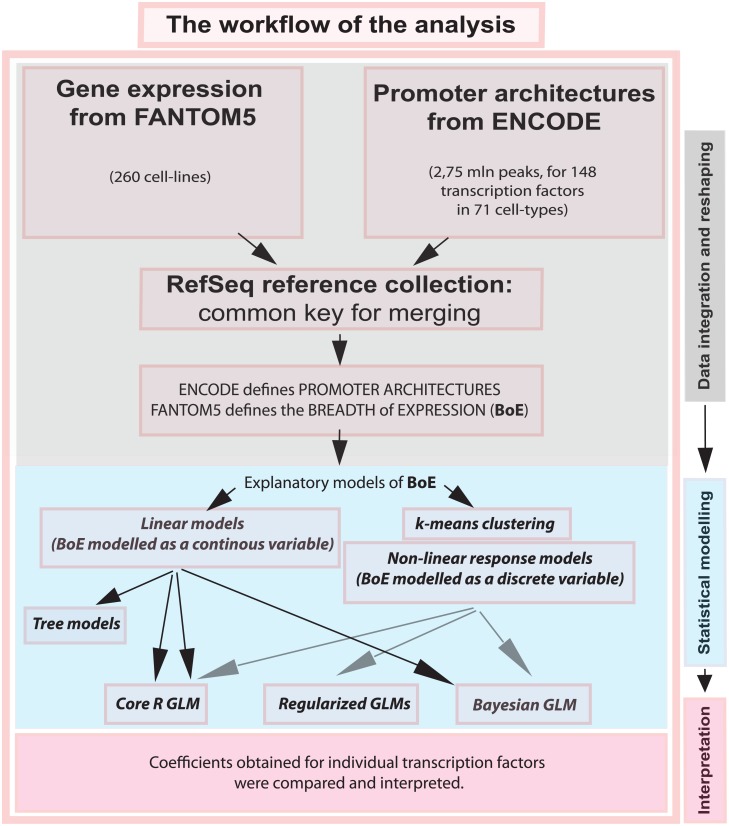
The workflow of data preparation and model building. In the first step, F5’s expression data were integrated with promoter architectures calculated from merged ENCODE ChIP-seq screens. Expression data were used to calculate BoE (BoE), that is the fraction of samples in which a transcript is expressed. In the second step, the integrated datasets were used to prepare model matrices and formulae. In the third step, tree models and GLMs were constructed and interpreted.

Herein, we apply several technologies of explanatory statistical modelling to the problem of modelling BoE from promoter architectures. (See the analysis workflow in Fig 1). We started with tree models for the initial description of the dataset [15]. Next, the core R implementation of GLMs was compared with regularization-based GLMs. The regularization methods included lasso, ridge and the elastic net. A Bayesian GLM was also constructed to provide an alternative to the above classical methods. Complementary non-linear-response models, were also constructed where *k*-means clustering was followed by multinomial regression to refine the response variable. Finally, TF coefficients calculated by different models were compared. In each case, the architectures of proximal promoters were the inputs for the models. BoE was the response variable which we sought to model and explain.

## Results

### How was BoE defined?

**Fig 2:**
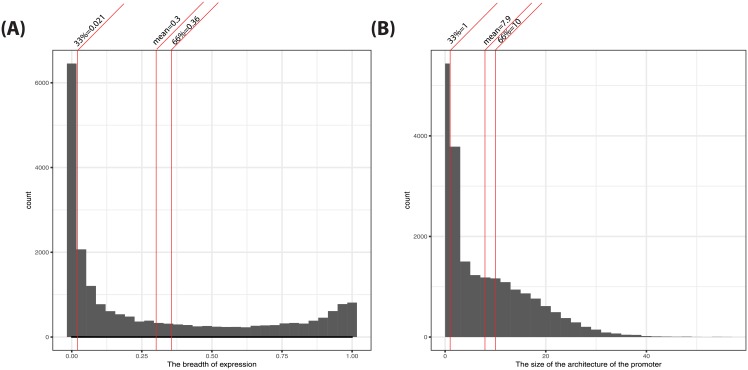
The distribution of BoE and the sizes of promoter architectures. The distribution of BoE (BoE), calculated from F5 expression data for the non-redundant set of transcripts, is shown in panel *A*. The distribution of the sizes of promoter architectures, computed from merged ENCODE data and sites with QS > 500, is shown in panel *B*. The non-redundant set of transcripts numbered 20,403. The breakdown of this total by chromosome was as follows: 2081, 1309, 1138, 779, 919, 1142, 946, 696, 801, 784, 1313, 1057, 343, 623, 580, 851, 1167, 303, 1423, 584, 236, 444, 858, and 26 on autosomes 1 through 22, and chromosome X and Y.

F5 expression data were in the format of tag counts from the technology of cap analysis of gene expression—CAGE [7]. To ensure comparability across different experiments, CAGE counts were normalized by the consortium to tags per million (TPM). BoE was defined as the fraction of tissues in which the transcript was expressed (that is present at more than 10 TPM, an accepted standard of the F5 consortium—alternative cut-offs were examined previously). The distribution of BoE is shown in Fig 2A. Also as reported previously [1], most transcripts were either narrowly (BoE < 0.33) or broadly (BoE > 0.66) expressed, with 65% and 23% of all transcripts in these categories, respectively. Transcripts with intermediate expression were under-represented (only 12%). Furthermore, the distribution of BoE was characterized by the minimum = 0 (n = 2,296), median = 0.11, mean = 0.3, maximum = 1 (n = 7), and the variance of 0.13.

### RefSeq transcripts were used as the common key for data integration

As before, the data on promoter architectures were integrated with BoE using RefSeq transcripts (S1 Dataset) as the common key for data merging [1]. After merging, there were 30,793 human RefSeq transcripts, and corresponding promoter architectures, for which F5 expression data were available. However, while we intentionally wanted to focus on transcripts rather than genes, multiple transcripts originating from the same promoter (that is from the same transcription start site—TSS) would be redundant having the same BoE and promoter architecture.

### How were the architectures of promoters defined?

The architectures of proximal promoters (S2 Dataset, redundant RefSeqs) were defined using the merged ENCODE data-set as described in detail previously [1]. The size of a promoter architecture was the cardinality of the corresponding multiset representation. In Fig 2B, we described the distribution of these sizes as a histogram. The distribution had the following characteristics: minimum = 0, median = 5, mean = 8, maximum = 58. Importantly, out of 20,403 non-redundant RefSeqs with expression data, 5,438 (27%) had no strong TF binding sites (TFBSes) in their proximal promoters (these are referred to as *empty architectures*). In total, there were 161,563 TFBSes (corresponding to ChIP-seq peaks) in all proximal promoters mapped to the non-redundant RefSeq transcripts.

### The motivation for: (1) sample choices, and (2) the boundaries of promoter size

It was already shown that the correlation between BoE and the size of promoter architecture held over all classes of F5 samples. The trend was also robust to the variation in the size of the window defining the proximal promoter. With robustness of the trend already demonstrated, for simplicity and clarity, this analysis did not split F5 samples into sub-categories but looked at the BoE calculated across all 1660 samples. In this dataset, there was a strong positive correlation between BoE and promoter size was the (Spearman’s *rho* = 0.68, *P*-value < 2e-16 for all data points, and *rho* = 0.52, *P*-value < 2e-16 after disregarding empty architectures). Moreover, just one size of the promoter window was chosen for our analysis. This was one kilobase window with symmetrical boundaries located ±500 bps from the TSS. We reasoned that the robustness of the trend has already been proven beyond any doubt. For this reason the inclusion of additional analysis variants and the associated detail would only obscure the main ideas, make the presentation difficult, and decrease readability.

### The motivation for the choice of ENCODE TF quality score (QS) cut-off

ENCODE TFBSes have a QS assigned to them which varied from zero through 1000. The consortium processed all separate ChIP-seq datasets through a unified bioinformatics pipeline [16] which assigned the score. The score reflected the confidence the consortium had in predicting a given site. In practice, high QS scores corresponded to a strong binding site in any single ENCODE sample and low scores corresponded to weak sites across ENCODE samples. Herein, we concentrated on strong ENCODE TFBSes, rather than on more numerous weak / low-occupancy binding sites whose functional significance is debatable [8, 17, 18].

### Dataset structure: ENCODE peaks for Pol II correlated with those for many TFs

In the ENCODE dataset, many TFs correlated strongly with Pol II. The top 10 Pol II-correlated TFs were as follows (Spearman’s *rhos* given in brackets): transcription initiation factor TFIID subunit 1—TAF1 (0.676), hairy/enhancer-of-split related with YRPW motif 1—HEY1 (0.549), nuclear factor kappa-light-chain-enhancer of activated B cells—NFKB (0.417), the Myc proto-oncogene—*c*-Myc (0.415), paired amphipathic helix protein Sin3a —Sin3Ak (0.411), TATA-Box Binding Protein—TBP (0.406), E74 like ETS TF 1—ELF1 (0.401), general transcription factor IIF subunit 1—GTF2F1 (0.357), E2F TF 1—E2F1 (0.339), and octamer transcription factor—Oct-2 (0.333). Perhaps not coincidentally these TFs also clustered together in the correlogram of TFs (S1 Fig).

Interestingly, three TFs had negative *rho* with Pol II suggesting they were inhibitory for housekeeping expression. These TFs were as follows (Spearman’s *rhos* given in brackets): polycomb repressive complex 2 subunit − SUZ12 (-0.04), neuron-restrictive silencer factor − NRSF (-0.02), and zinc finger protein 274 − ZNF274 (-0.002).

TFs could also correlate with each other, especially if they belonged to the same gene family (Table 1, S1 Table). Statistical significance of the correlations was established using an asymptotic approximation to the exact test, as well as a permutation procedure (permutation tests are more appropriate to genomic data which are not a sample from a population). The random genomic expectation was obtained by the permutation of the sites transcriptome-wide a sufficient number of times (in this case, n = 10). Each time, Spearman’s *rhos* were recalculated for the entire permuted set. This random expectation was used to define significance thresholds corresponding to critical values in a two-sided hypothesis test at 5% significance level: -0.0104 and 0.0124. A plot visualizing the random expectation, the observed distribution of correlations in the ENCODE dataset, and the determined thresholds is given in S2 Fig: “Random expectation and the observed distribution of Spearman’s correlation coefficients”.

**Table 1:** The correlated pairs of TFs. All the correlations shown were highly statistically significant (*P*-value < 0.0001). Positive correlations between TFs were of much greater absolute magnitude than negative (the maximum *rho* was 0.99, the minimum was only *-0*.*095*). However, the negative correlations were still statistically significant (S1 Table).

TF1	TF2	*rho*
Pol3	RPC155	0.759
FOXA1	FOXA2	0.76
BRCA1	ZBTB33	0.762
MafF (M8194)	MafK (ab50322)	0.774
BDP1	Pol3	0.79
USF1 (sc8983)	USF2	0.793
AP2alpha	AP-2gamma	0.822
NF-YA	NF-YB	0.831
BRF1	RPC155	0.832
BRF1	Pol3	0.852
USF1	USF1 (sc8983)	0.854
BDP1	BRF1	0.866
YY1	YY1 (c20)	0.898
BDP1	RPC155	0.961
Oct-2	POU2F2	0.995

NOTE: Thirty pairs with the highest correlation coefficients are shown. The full table is given as S1 Table. In S1 Fig, correlated TF clusters were visualized. To confirm the statistical significance of TF correlations, a permutation analysis of the TFs peaks transcriptome-wide was performed. The random expectation for the distribution of Spearman’s *rhos* (S2 Fig) was calculated. The null hypothesis was that a given *rho* value derived from the random distribution. Based on the randomizations, the 5% rejection region for the null hypothesis was determined. The region consisted of the union of two intervals: [-1, -0.012) and (0.015, 1]. Additionally, an approximation of an exact *P*-value was computed for each correlation test asymptotically (S1 Table).

### Multicollinearity

Highly correlated pairs of TFs could be a problem for the modelling strategy adopted here as multiple regression produces correlation coefficients *after* controlling for all the other covariates. This is otherwise known as the multicollinearity problem. To control the problem, the variance inflation factor (VIF) was calculated for all TFs. The recommendation in the literature is to remove variables with VIF higher than ten [19]. The TFs with the VIF higher than five were as follows: octamer TF 2 − Oct-2 (VIF = 95.4), POU class 2 homeobox 2 − POU2F2 (95.2), BDP1 (19.5), RPC155 (16.1), BRF1 (10.5), USF1 (5.7), Pol III (5.6), and YY1 (5.4). These inflated VIFs were due to the highly correlated TF pairs. As a remedy, the following variables were removed from the *P* matrix: Oct-2, BDP1, upstream TF 1 − USF1 (the sc8983 antibody), RPC155, and YY1 (the c20 antibody). After this selection, there was only one variable with VIF > 5 (namely BRF1 with VIF = 5.2).

### Modelling philosophy

In an influential review, Galit Shmueli [13] stressed that the strategy of explanatory modelling must be suggested by prior theoretical knowledge and scientific insights about the natural phenomenon under investigation. What informed the modelling process in this work was the theoretical knowledge of the biochemical steps through which TFs bind DNA to open the chromatin, attract RNA polymerases, and activate transcription. One of the key decisions was that Pol II sites were not included as explanatory variables for multiple regression. (ChIP-seq peaks identified by four antibodies targeting Pol II from the inputs of GLMs were removed.) We reasoned that including them would be a methodological mistake. As suggested by Gelman *et al*. one must not control for the consequences of the activation of the process being modelled [20].

### The model matrix and model formulae

Model inputs were the architectures of the promoters which defined an *N* x *M* matrix *P*. *N* was the number of TFs in the ENCODE dataset (*N* = 148). *M* was the number of RefSeq transcripts. Thus, the input variables for the models were counts of individual TFBSes per proximal promoter. The *P* matrix was sparse (95% values were zeros). Note, however, that *N* equalled 144 after the removal of four polymerase type II associated variables. *N* equalled 138 after the removal of highly correlated TFs.

### Binary partitioning trees

Tree models were used to identify the top sources of variation in BoE among TFBSes. The tree algorithm works by binary partitioning [21]. Such tree models [15] are useful for the initial inspection of datasets with multivariate regression problems and in such a role they were applied here.

**Fig 3:**
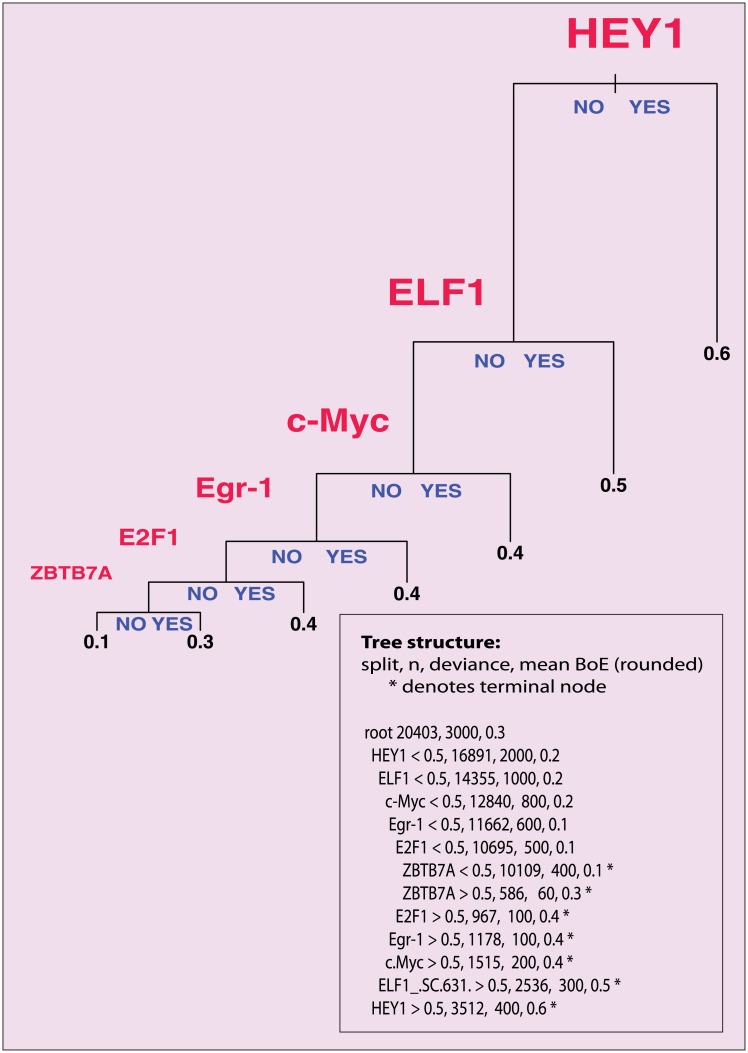
A tree model of BoE. The model was fitted using binary recursive partitioning [15]. This simple figure gives intuitive insight into the transcriptions factors which had the greatest impact on BoE. Each path from the top of the tree to one of the terminal leaves at the bottom follows through a series of splits. At each split, one follows a YES or NO direction according to whether a given TF is present in or absent from the promoter architecture. At each terminal leaf, the mean BoE for a given subset of the data is given. The TFs with the most significant contributions to the reduction in deviance are as follows: HEY1, ELF1, *c*-Myc, Egr-1, E2F1, and ZBTB7A. Additionally, at the bottom of each panel, the tree structure is given in text with the number of transcripts (n), deviance, and mean BoE for each group.

The first tree (S3 Fig) included all explanatory variables. In this tree, RNA polymerase type II (Pol II), TAF1 and phosphorylated Pol II were chosen by the algorithm as the first, the second, and the third partitioning variables, in this order of importance. However, these associations were trivial and Pol II, which is not a TF, could mask more meaningful associations. Therefore, in the second tree (Fig 3), Pol II, TAF1 and phosphorylated Pol II covariates were removed to reveal the deeper structure of the dataset. The second tree identified the following partitioning variables (that is TFs), in the descending order of importance: (1) HEY1 [22], (2) ELF1 [23], (3) *c*-Myc, (4) early growth response protein 1 − Egr-1 [24], (5) E2F1 [25], and (6) zinc finger and BTB domain-containing protein 7A − ZBTB7A. For each variable the value of 0.5 was reported to be the cutoff making the splits “yes or no” (presence or absence) decisions for respective TFBSes.

### GLMs

The tree model was useful in defining the top sources of variability in the dataset. However, unlike regression, the tree model does not produce the estimates of correlation coefficients for all significant TFs. Instead, this was achieved by fitting GLMs [14, 26]. The motivation for using GLMs was that BoE was conveniently interpreted as a proportion with a binomial structure of errors. Consequently, the residuals were not normally distributed. This violated the assumptions of linear regression based on the sum of least squares [21]. Here, we started by using the *glm()* function of the core R [27]. The coefficients for individual TFs obtained by this model are shown in S2 Table. The coefficients of different GLMs are summarized in Table 2 and fully listed in S3 Table.

**Table 2:** The comparison of coefficients of different GLMs. TFs with negative coefficients are highlighted in italics.

Term	Linear-response					Non-linear-response					Median
	GLM	Regularized GLM			STAN	GLM	Regularized GLM			STAN	
		Lasso	Ridge	Elastic net			Lasso	Ridge	Elastic net		
*SUZ12*	*-0*.*6*	*-0*.*6*	*-0*.*5*	*-0*.*6*	*-0*.*8*	*-1*.*3*	*-0*.*7*	*-0*.*5*	*-0*.*9*	*-1*.*6*	*-0*.*7*
*NRSF*	*-0*.*5*	*-0*.*5*	*-0*.*4*	*-0*.*5*	*-0*.*8*	*-0*.*7*	*-0*.*2*	*-0*.*5*	*-0*.*3*	*-0*.*7*	*-0*.*5*
*ZNF274*	*-9*.*3*	*-1*.*4*	*-1*.*5*	*-0*.*3*	*3*.*3*	*-7*	*0*	*-0*.*3*	*0*	*2*.*5*	*-0*.*3*
*MEF2A*	*-0*.*3*	*-0*.*3*	*-0*.*3*	*-0*.*3*	*-0*.*8*	*-0*.*6*	*0*	*0*.*1*	*0*	*-0*.*6*	*-0*.*3*
*TAL1*	*-0*.*3*	*-0*.*3*	*-0*.*2*	*-0*.*2*	*-3*.*6*	*-0*.*7*	*0*	*-0*.*5*	*-0*.*2*	*-1*	*-0*.*3*
*HNF4A*	*-0*.*2*	*-0*.*2*	*-0*.*2*	*-0*.*2*	*-0*.*8*	*-0*.*6*	*0*	*-0*.*2*	*-0*.*1*	*-0*.*8*	*-0*.*2*
*GRp20*	*-0*.*2*	*-0*.*2*	*-0*.*2*	*-0*.*2*	*-0*.*6*	*-0*.*7*	*0*	*0*	*0*	*-1*.*4*	*-0*.*2*
*BCL11A*	*-0*.*2*	*-0*.*2*	*-0*.*2*	*-0*.*2*	*-1*.*3*	*-0*.*2*	*0*	*-0*.*2*	*0*	*-1*.*2*	*-0*.*2*
*IRF4*	*-0*.*2*	*-0*.*2*	*-0*.*2*	*-0*.*2*	*-0*.*6*	*-0*.*7*	*0*	*0*	*0*	*-0*.*8*	*-0*.*2*
*CTCF(N)*	*-0*.*2*	*-0*.*2*	*-0*.*2*	*-0*.*2*	*-0*.*4*	*-0*.*6*	*0*	*-0*.*2*	*-0*.*1*	*-0*.*6*	*-0*.*2*
*FOS*	*-0*.*2*	*-0*.*2*	*-0*.*2*	*-0*.*1*	*-0*.*5*	*-0*.*3*	*0*	*0*.*1*	*0*	*-0*.*9*	*-0*.*2*
*GATA3*	*-0*.*2*	*-0*.*2*	*-0*.*2*	*-0*.*2*	*-0*.*5*	*-0*.*4*	*0*	*0*	*0*	*-0*.*6*	*-0*.*2*
*FOXA1*	*-0*.*2*	*-0*.*1*	*-0*.*1*	*-0*.*1*	*-0*.*7*	*-0*.*6*	*0*	*-0*.*2*	*0*	*-0*.*9*	*-0*.*2*
*Rad21*	*-0*.*1*	*-0*.*1*	*-0*.*1*	*-0*.*1*	*-0*.*3*	*-0*.*3*	*0*	*-0*.*1*	*0*	*-0*.*4*	*-0*.*1*
*cJun*	*-0*.*1*	*-0*.*1*	*-0*.*1*	*-0*.*1*	*-0*.*5*	*-0*.*4*	*0*	*-0*.*2*	*0*	*-0*.*5*	*-0*.*1*
*GCN5*	*-0*.*1*	*-0*.*1*	*-0*.*1*	*-0*.*1*	*-5*.*4*	*-0*.*4*	*0*	*0*.*3*	*0*	*5*.*9*	*-0*.*1*
*PGC1A*	*-0*.*1*	*-0*.*1*	*-0*.*1*	*-0*.*1*	*-5*.*1*	*-0*.*6*	*0*	*0*	*0*	*0*.*5*	*-0*.*1*
*GATA2*	*-0*.*1*	*-0*.*1*	*-0*.*1*	*-0*.*1*	*-0*.*6*	*-0*.*3*	*0*	*0*	*0*	*-0*.*6*	*-0*.*1*
*GTF2B*	*-0*.*1*	*-0*.*1*	*-0*.*1*	*0*	*-1*.*4*	*-0*.*1*	*0*	*0*.*2*	*0*.*1*	*-0*.*5*	*-0*.*1*
*Brg1*	*-0*.*1*	*-0*.*1*	*-0*.*1*	*-0*.*1*	*-0*.*3*	*-0*.*1*	*0*	*0*.*2*	*0*	*-0*.*2*	*-0*.*1*
*RFX5*	*-0*.*1*	*-0*.*1*	*-0*.*1*	*0*	*-5*.*4*	*-0*.*5*	*0*	*-0*.*1*	*0*	*-1*.*3*	*-0*.*1*
*p300*	*0*	*0*	*0*	*0*	*-0*.*5*	*-0*.*3*	*0*	*-0*.*2*	*-0*.*1*	*-1*	*-0*.*1*
*BRF2*	*-0*.*1*	*-0*.*1*	*-0*.*1*	*0*	*-8*.*1*	*-0*.*8*	*0*	*-0*.*1*	*0*	*4*.*5*	*-0*.*1*
*THAP1*	*-0*.*1*	*-0*.*1*	*0*	*-0*.*1*	*-0*.*4*	*-0*.*4*	*0*	*0*	*0*	*-0*.*5*	*-0*.*1*
*TCF12*	*-0*.*1*	*-0*.*1*	*-0*.*1*	*-0*.*1*	*-0*.*2*	*-0*.*2*	*0*	*0*	*0*	*-0*.*2*	*-0*.*1*
*E2F4*	*-0*.*1*	*-0*.*1*	*-0*.*1*	*-0*.*1*	*-0*.*3*	*0*	*0*	*0*.*2*	*0*	*0*	*-0*.*1*
*GATA*.*1*	*-0*.*1*	*0*	*0*	*0*	*-0*.*3*	*-0*.*2*	*0*	*0*	*0*	*-0*.*3*	*0*
*SETDB1*	*0*	*0*	*0*	*0*	*-0*.*2*	*-0*.*2*	*0*	*0*	*0*	*-0*.*2*	*0*
*Pol3*	*-0*.*1*	*-0*.*1*	*-0*.*1*	*0*	*-2*.*9*	*0*.*4*	*0*	*0*.*2*	*0*	*-0*.*4*	*0*
*USF2*	*-0*.*1*	*-0*.*1*	*0*	*0*	*-0*.*1*	*0*	*0*	*0*.*1*	*0*	*0*	*0*
HDAC8	0.2	0.1	0.1	0	13.5	0.4	0.1	0.6	0.3	-13.7	0.1
cMyc	0.1	0.1	0.1	0.1	0.4	0.4	0.2	0.3	0.2	0.4	0.2
JunD	0.2	0.2	0.1	0.1	0.3	0.4	0.1	0.2	0.1	0.4	0.2
NFKB	0.2	0.2	0.2	0.2	0.4	0.4	0.1	0.4	0.1	0.4	0.2
ZNF263	0.2	0.2	0.2	0.2	0.3	0.2	0	0.1	0	0.1	0.2
HSF1	0	0	0	0	-1.1	0.7	0.4	0.4	0.5	9.9	0.2
Ini1	0.2	0.2	0.2	0.2	0.4	0.4	0	0.3	0.1	0.5	0.2
USF1	0.2	0.2	0.1	0.1	0.3	0.2	0	0.2	0	0.3	0.2
Egr1	0.2	0.2	0.2	0.2	0.3	0.4	0	0.2	0	0.4	0.2
ZBTB7A	0.2	0.2	0.2	0.2	0.3	0.3	0	0.2	0	0.3	0.2
CCNT2	0.2	0.2	0.2	0.2	0.3	0.5	0.2	0.4	0.2	0.5	0.2
ZZZ3	0.3	0.2	0.2	0.2	-2.1	0.4	0	0.2	0	3.3	0.2
GABP	0.2	0.2	0.2	0.2	0.4	0.5	0.1	0.3	0.1	0.6	0.2
CTCF(C)	0.2	0.2	0.2	0.2	0.5	0.6	0	0.3	0.1	0.7	0.2
Nrf1	0.2	0.2	0.2	0.2	0.5	0.6	0.1	0.4	0.1	0.6	0.2
BRCA1	0.2	0.2	0.1	0.1	0.4	0.6	0.2	0.4	0.3	0.8	0.2
POU2F2	0	0	0	0	0.3	0.3	0.2	0.4	0.2	0.6	0.2
XRCC4	0.2	0.2	0.2	0.1	8.2	1.4	0	0.8	0.2	8.4	0.2
GTF2F1	0.1	0.1	0.1	0.1	0.4	0.4	0.2	0.4	0.2	0.6	0.2
ELF1	0.2	0.2	0.2	0.2	0.4	0.4	0	0.3	0	0.5	0.2
ERRA	0.2	0.2	0.2	0.2	4.6	0.7	0.2	0.4	0.3	0.8	0.2
STAT1	0.3	0.2	0.2	0.2	1.2	0.5	0.2	0.3	0.3	1	0.3
YY1	0.3	0.3	0.3	0.3	0.8	0.8	0.2	0.6	0.2	0.9	0.3
TAF7	0	0	0	0	1.1	0.3	0.3	0.4	0.4	1.1	0.3
HA-E2F1	0.3	0.3	0.3	0.3	0.6	0.7	0.2	0.4	0.2	0.8	0.3
HEY1	0.3	0.3	0.3	0.3	0.7	0.8	0.3	0.6	0.4	0.9	0.3
SREBP1	0.2	0.2	0.2	0.2	0.7	0.6	0.3	0.4	0.4	1.7	0.4
IRF3	0.1	0.1	0.1	0.1	0.5	0.5	0.4	0.4	0.5	0.6	0.4
TFIIIC-110	0.4	0.4	0.4	0.4	0.9	0.9	0.4	0.5	0.4	0.9	0.4
SPT20	0.3	0.3	0.2	0.2	-4.5	1.5	0.7	1	0.8	5.4	0.5

### Regularized GLMs

GLMs can suffer from the problems of multicollinearity and over-fitting to which shrinkage and regularization methods are a remedy [28]. Here, we used *glmnet*, which is a popular R package implementing GLMs with regularization and variable selection. Elastic net regularization [29] performed by the package is an even more effective variable selection mechanism than the original lasso method [30], or ridge, which *glmnet* generalizes.

Lasso, ridge and the elastic net are alternative shrinkage methods in which correlation coefficients of multiple regression are shrunk towards each other and towards zero to prevent over-fitting, and to select covariates which are the most relevant for the model [28]. The lasso was designed to select a single variable from a correlated group if they are collinear (eliminating others). Ridge shrinks such coefficients towards each other and towards zero. Ridge and lasso were compared during a meta-analysis of lung cancer gene expression datasets [31]. The lasso penalty resulted in a classifier which included fewer genes, but had similar predictive performance to the ridge model.

### Activating, saturating, and inhibitory regulatory logic of TFs

**Fig 4:**
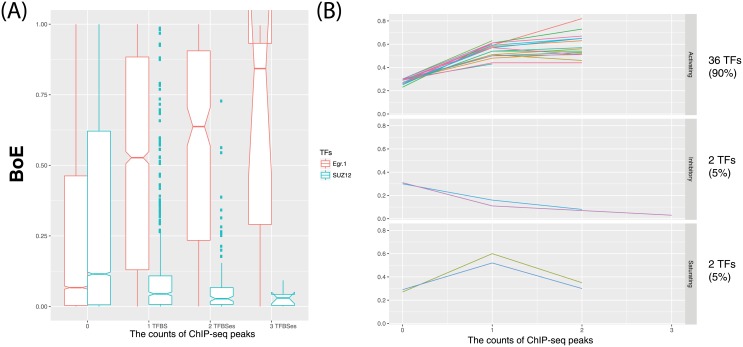
The activating, inhibitory, and saturating regulatory logic of TFs. In panel A, the boxplot of BoE depending on the number of binding sites is shown for two TF examples: Egr-1 and SUZ12. The first is activating for BoE, the second is inhibitory. In Panel B, the average BoE depending on the number of binding sites (with more than three genes in each group) is shown for 40 TFs identified by regression as the most significant contributors to the regulation of BoE. This shows that a distinction between activating and inhibitory TFs can be applied more generally. In addition, a small third category of saturating TFs could be identified. However, the overwhelming majority of the TFs (90%) were classified as activating.

In Fig 4A, two contrasting examples of TFs and of their impact on BoE are shown. The first TF, Egr-1, is an example of the activating regulatory logic. For this TF the average BoE of transcripts increases with each additional binding site. (In other words, BoE is the function of the number of TFBSes—TFBSes, that is monotonically increasing.) In contrast, SUZ12 is an example of inhibitory logic. The average BoE of transcripts decreases with each additional binding site. The differences between the effects of the above TFs were startling: transcripts with three Egr-1 promoter binding sites had the average BoE of 0.61 (N = 7). For SUZ12, this value was only 0.032 (N = 9) that is 19 times lower (Wilcoxon test P-value = 0.02).

In addition to activating and inhibitory TFs, a third category of saturating TFs could be identified (Fig 4B). For saturating TFs, the average BoE was higher for genes with one TFBS than for those without any, but genes with two binding sites had statistically significantly lower BoE than those with a single site (Wilcoxon test). In other words, for saturating TFs the relationship between BoE and the number of TFBSes was non-linear (and the corresponding function was not monotonic).

We applied the above classification scheme to forty selected TFs which were found to have regression coefficients statistically significantly different from zero. (If all TFs were included, due to multicollinearity, TFs simply associating with BoE rather than causative would be identified). In the group of these TFs, 36 were classified as activating, 2 as inhibitory (NRSF and SUZ12), and 2 as saturating (GABP and Pbx3). The list of the average values of BoE for the selected TFs is given in Table 3.

**Table 3:** Three types of regulatory logic. Mean BoE (and the number of transcripts) are given in the four columns corresponding to 0, 1, 2 and 3 TFBSes of a given type.

TF	No TFBSes	1 TFBS	2 TFBSes	3 TFBSes	Effect
AP-2gamma	0.29(19147)	0.5(1242)	0.57(14)	NA	Activating
ATF3	0.29(19606)	0.59(790)	0.82(7)	NA	
BRCA1	0.29(19891)	0.64(511)	0.97(1)	NA	
CCNT2	0.26(17770)	0.58(2574)	0.63(59)	NA	
CEBPB	0.29(19309)	0.48(1071)	0.52(22)	0(1)	
cMyc	0.23(16095)	0.56(3824)	0.63(465)	0.71(19)	
CTCF(C)	0.29(19172)	0.47(1209)	0.47(21)	0.88(1)	
CTCF(N)	0.3(19668)	0.39(724)	0.24(11)	NA	
E2F4	0.29(19388)	0.51(1001)	0.56(14)	NA	
E2F6	0.29(18968)	0.51(1404)	0.46(31)	NA	
Egr1	0.26(17067)	0.51(3085)	0.57(244)	0.61(7)	
ELF1	0.24(16351)	0.56(3940)	0.62(112)	NA	
GATA3	0.3(20171)	0.41(231)	0(1)	NA	
GRp20	0.3(20274)	0.63(129)	NA(NA)	NA	
GTF2F1	0.28(19162)	0.65(1234)	0.54(7)	NA	
HA-E2F1	0.25(16496)	0.52(3750)	0.62(153)	0.6(4)	
HEY1	0.23(16891)	0.61(3187)	0.73(324)	0.11(1)	
HNF4A	0.3(19960)	0.33(438)	0.1(5)	NA	
Ini1	0.26(17625)	0.54(2621)	0.54(155)	0.74(2)	
IRF4	0.3(20118)	0.47(283)	0.05(2)	NA	
JunD	0.29(19402)	0.5(978)	0.53(23)	NA	
Max	0.26(17463)	0.54(2847)	0.57(90)	0.41(3)	
MEF2A	0.3(20211)	0.43(189)	0.07(3)	NA	
NFKB	0.25(17292)	0.59(2974)	0.65(135)	0.48(2)	
Nrf1	0.27(18455)	0.57(1906)	0.65(42)	NA	
SREBP1	0.3(20227)	0.61(176)	NA(NA)	NA	
STAT1	0.3(20138)	0.5(260)	0.51(5)	NA	
TFIIIC-110	0.3(20260)	0.61(142)	0.98(1)	NA	
TR4	0.29(19781)	0.6(622)	NA(NA)	NA	
USF1	0.28(18848)	0.54(1536)	0.49(19)	NA	
USF2	0.29(19450)	0.57(943)	0.51(10)	NA	
YY1	0.26(18121)	0.61(2216)	0.67(65)	0.58(1)	
ZBTB7A	0.27(17289)	0.5(2749)	0.53(347)	0.44(18)	
ZEB1	0.29(19368)	0.49(1016)	0.37(19)	NA	
Znf143	0.28(18974)	0.53(1395)	0.47(34)	NA	
ZNF263	0.29(18818)	0.44(1569)	0.44(16)	NA	
NRSF	0.3(20190)	0.16(209)	0.08(4)	NA	Inhibitory
SUZ12	0.31(19753)	0.11(533)	0.07(108)	0.03(9)	
GABP	0.27(18468)	0.6(1925)	0.35(10)	NA	Saturating
Pbx3	0.29(19573)	0.52(803)	0.3(27)	NA	

### The interactions between TFs

**Fig 5:**
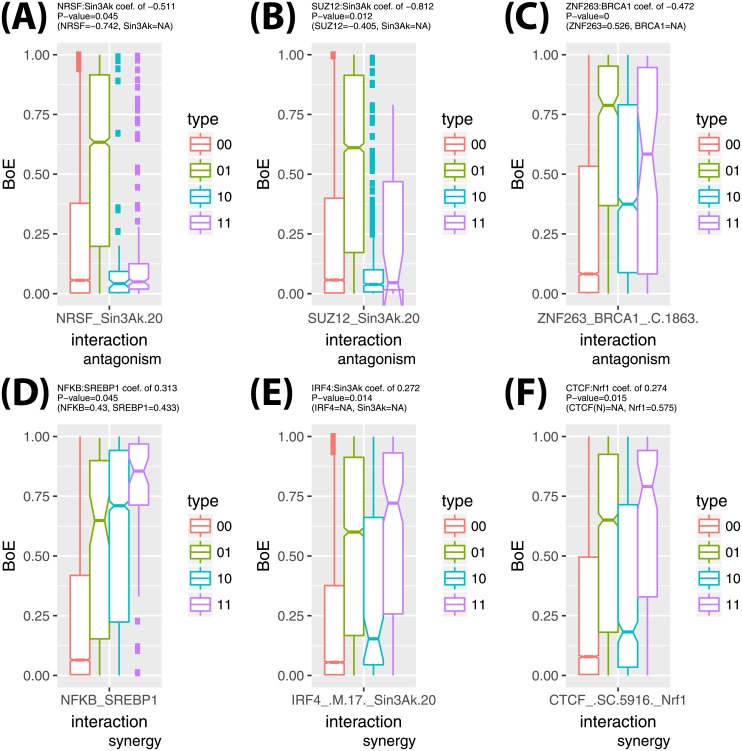
The examples of TF interactions. Six examples of the interactions of TFs in their impact on BoE are illustrated in panels A to F. Above each panel, the coefficients of regression (and their P-values) for the interaction term and first-order terms are given if they are statistically significant (*NA* indicates the first-order coefficient was not significant). Each panel contains a boxplot of BoE with hinges for four types of promoter architectures: without any of the sites of the pair (highlighted in red), with the second interaction term only (green), with the first interaction term only (blue), and with both interaction terms (violet).

Multiple regression can detect statistical interactions between model terms. Such interactions suggest the antagonism or synergy of the impact of pairs of TFs on BoE. When pairwise interactions between terms were modelled as an extension of the core R GLM linear model, there were 110 interaction pairs at the P-value cut-off of 0.05. Several TFs were frequently included in these interaction pairs, for example: ELF1 (in 13 pairs), ZBTB7A (12 pairs), Nrf1 (12 pairs), HEY1 (12 pairs), Egr-1 (11 pairs), NFKB (10 pairs), Ini1 (10 pairs), or E2F1 (10 pairs). In Fig 5, six examples of TF interactions and their impact on BoE are shown. Panels A and B, illustrate the attenuating impact of inhibiting TFs (NRSF and SUZ12) on Sin3Ak. Panel C illustrates the antagonism of the ZNF263 and BRCA interaction. Finally, panels D, E and F suggest synergy between the following three pairs of TFs: NFKB:SREBP1, IRF4:Sin3Ak, and Nrf1:CTCF(C).

### *K*-means clustering and non-linear-response models

**Fig 6:**
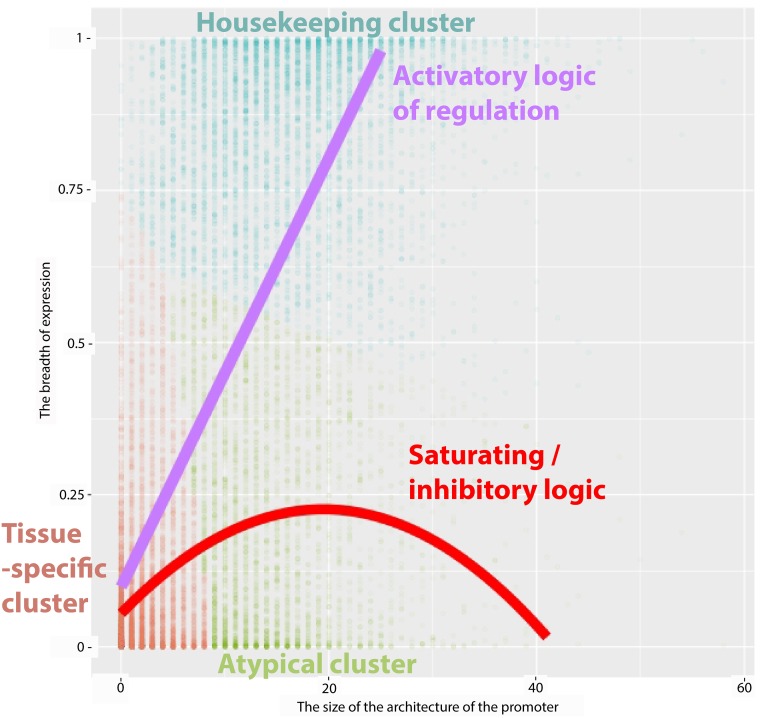
Three clusters of transcripts. The *k*-means algorithm clustered transcripts into three classes with regards to the size of promoter architecture and BoE (these clusters are labeled *tissue-specific*, *housekeeping*, and *atypical*). The X-axis corresponds to the size of promoter architectures. The Y-axis corresponds to BoE. The housekeeping cluster and the violet line of a linear model (intercept = 0.09, regression coefficient = 0.035, *P* < 2e-16) illustrate the activating logic of pro-housekeeping TFs. In contrast, the atypical cluster and the red line of a quadratic linear model (intercept = 0.055, coefficients of 0.0176 and -0.00045 for the first order and quadratic terms respectively, *P* < 2e-16) illustrate the inhibitory logic of pro-tissue-specific TFs.

As a refinement of GLMs, non-linear-response modeling of BoE was pursued. In the first step, *K*-means clustering (a non-linear classification algorithm) was used to define three groups of transcripts with respect to BoE and the sizes of their promoter architectures (Table 4). These three clusters were visualized in Fig 6. The *tissue-specific* cluster consisted of transcripts with small promoter architectures (n = 11,176). The *housekeeping* cluster included broadly expressed transcripts with large promoter architectures (n = 5,299). The *atypical* cluster consisted of transcripts without the positive correlation between the architecture size and BoE (n = 3,928). In the second step, the above three cluster were used to encode a response variable for regularized *multinomial* regression implemented in the *glmnet* package. Coefficients from non-linear-response models were compared with those of linear response models in Table 2.

**Table 4:** Three clusters of transcripts. Three clusters of transcripts were identified through *k*-means clustering (Fig 6) and labelled I, II and III. Of these, II was the cluster of housekeeping transcripts. The average BoE of this cluster was 0.84, that is 7.6 fold higher than the value of 0.11 obtained for the remaining transcripts (Wilcoxon text, *P*-value < 2e-16). Transcripts in cluster II had also greater average size of promoter architecture: 16 versus 5 for others (Wilcoxon text, *P*-value < 2e-16).

Cluster	BoE, *F* = 55784, *P* < 2e-16*	The average size of promoter architecture			
		Raw count, *F* = 17833, *P* < 2e-16	Unique sites, *F* = 18693, *P* < 2e-16	No Pol II sites, *F* = 16393, *P* < 2e-16	Unique sites and no Pol II, *F* = 17123, *P* < 2e-16
TISSUE-SPECIFIC	0.07 (0.02 **)	1.62 (4.64)	1.58 (4.44)	1.41 (3.76)	1.38 (3.61)
HOUSEKEEPING	0.84 (0.02)	16.29 (68.11)	15.44 (57.17)	14.51 (59.79)	13.81 (51.02)
ATYPICAL	0.21 (0.03)	14.55 (38.90)	13.92 (33.59)	13.15 (34.82)	12.64 (30.57)

* The values of the *F* statistic and *P*-values for each column derive from the analysis of variance (ANOVA).

** The numbers in curly brackets signify variance.

### Random forest

Variable importance (S4 Table) was measured using percentage increase in mean squared error when variable was omitted. The distribution of variable importance was right-skewed. The minimum, median, mean and maximum scores were -7, 15, 19.8 and 114. The eleven TFs with scores higher than 50 were as follows: HEY1, ZBTB7A, Egr1, YY1, Ini1, HA-E2F1, cMyc, ELF1, Nrf1, HMGN3 and NFKB. Random forest’s variable importance scores can not be directly compared correlation coefficients as importance scores can be high for TFs both positively and negatively correlated with housekeeping expression. However, ten out of the eleven most important TFs were positively correlated with housekeeping expression in the GLMs of Table 2.

### The pairs of antibodies targeting the same protein

The ENCODE dataset contains several pairs of antibodies targeting the same protein [32]. It is interesting to compare the results obtained for these pairs. For example, there were two antibodies targeting USF-1 (sc-229 and sc-8983). Their peaks correlated highly with each other (*rho* = 0.862). There were also two antibodies targeting YY1: sc-1703 and sc-281, also highly correlated (*rho* = 0.896). Two antibodies targeting MAF BZIP TF K − MafK (ab50322 and sc-477) correlated at *rho* = 0.772. In conclusion, the results from the pairs of antibodies were largely in agreement.

The exception to the above rule were two antibodies targeting CCCTC-binding factor − CTCF, antibody C-20 (sc-15914) and N-17 (sc-5916). Both were goat polyclonal antibodies [32]. While these antibodies correlated (*rho* = 0.618), the first was found to have a positive impact on BoE, while the second had negative impact. We note that the first antibody targets the epitope near the C-terminus of human CTCF—denoted as CTCF(C), while the second targets N-terminus of the protein—denoted as CTCF(N). It is likely that these termini have different reactivity profiles in ChIP-seq assays.

### The subset of dual Pol II-Pol III promoters

One of the interesting sub-groups of transcripts were 34 housekeeping transcripts arising from dual Pol II-Pol III promoters. (Typically, Pol III promoters are associated with rRNAs and tRNAs genes [33]. However, such transcripts were excluded in the F5 pipeline.) The dual promoters which *were* included in the pipeline were not pure Pol III promoters. In fact, they were enriched in Pol II in addition to having a Pol III ChIP-seq peak. Only two out of the 34 did not have a Pol II peak, versus 13,846 out of 30,759 (*P*-value = 0.0000008, Fisher’s exact test, giving an enrichment ratio of 13).

Reassuringly, Pol III peaks correlated positively with those for several known co-factors of RNA polymerase III. The correlated TFs were as follows (those with *rho* > 0.1): RNA polymerase III transcription initiation factor—BRF1 (*rho* = 0.852), transcription initiation factor IIIB—BDP1 (0.79), the catalytic core and largest (155kDa) component of Pol III − RPC155 (0.759), heat shock factor 1 − HSF1 (0.417), TF for polymerase III C − TFIIIC (0.301), histone acetyltransferase p300—N-15 rabbit polyclonal antibody (0.297), and TATA-binding protein − TBP (0.147).

### The effect of the level of gene expression

We [1] and others [5] reported before that BoE correlated with the average level of gene expression. In the dataset used here, there was a Spearman correlation of *rho* = 0.96 between BoE and mean expression, and *rho* = 0.9 with the median. The correlations persisted even if the average or median expression were calculated only across tissues in which a gene was expressed: *rhos* of 0.37 and 0.41, respectively. (*P*-values for all the above correlations were lower than 2.2e-16.)

To ensure that our statistical models were specific to BoE, we verified that the effects of individual TFs on BoE persisted even after controlling for the level of gene expression. If mean, median, or maximal expression were included as explanatory variables, we obtained similar models to those obtained with default inputs. This was evident from correlations between coefficient vectors for default inputs and for inputs including mean (*rho* = 0.93), median (*rho* = 0.95), or maximal expression (*rho* = 0.97) as covariates. (At the same time, models with average or median expression as the response were only weakly similar with *rhos* of 0.3 and 0.43, respectively.)

## Discussion

Herein, we have integrated F5 and ENCODE and sought to identify pro-housekeeping and pro-tissue-specific TFBSes in proximal promoters of human transcripts. Such a strategy of integrative bioinformatics can help reveal genome-wide trends that, as essentially statistical arguments, could not be identified by the study of individual loci or verified experimentally using the techniques of molecular biology.

The exceptionally comprehensive functional genomic resources of F5 and ENCODE were intentionally integrated using the RefSeq reference transcript collection. We argue that TSSes of experimentally-verified transcripts, rather than predicted gene models or alternative splice forms, are the natural focus for this analysis. Not only are the original expression data but also ENCODE data mapped to TSSes. One of the key findings of F5 was that many genes have multiple TSSes which differ strongly in their expression patterns [7]. For the obvious reasons of alternative genomic location, these alternative TSSes also differ in the architectures of their proximal promoters. All the above considerations prompted us to deliberately and purposefully focus on the set of non-redundant TSSes linked to RefSeq transcripts and to avoid the focus on genes. (We note, however, that the correlation between promoter sizes and BoE was previously shown to exist both on the level of transcripts and on the level of genes [1, 9].)

We began the analysis with a tree model (S3 Fig), which stratified the dataset into four sub-sets of TSSes with increasing average BoE (increasing gradually from 0.08 through 0.4, 0.5 to 0.7). This stratification depended on the presence of promoter ChIP-seq peaks of RNA polymerase II (Pol II), TAF1, and S2-phosphorylated Pol II. TAF1, or TFIID subunit 1, is otherwise known as the TATA-box binding protein associated factor 1. TAF1 interacts with the TATA-box binding protein (TBP) and other co-factors in forming the Pol II initiation complex; it is one of basal factors required for Pol II transcription initiation [34]. It was reassuring that so much variability in the data could be explained just by these basic components of the polymerase II complex. After all, polymerase II is responsible for the transcription of messenger RNAs of the majority of genes—protein coding genes. However, the presence of these factors does not explain the mechanism of the activation of expression: it is merely a consequence of it.

We note that some of the polymerase II peaks, especially those detected by the non-phospho-specific antibody labelled *Pol II* [35], might be bound to proximal promoter but in a paused state [36]. However, the S2-phospho-specific antibody − ab5095 [37] recognized phosphorylated polymerase. (Phosphorylated in the amino-acid position two of its C-terminal repeat YSPTSPS.) Importantly, this phosphorylated form is thought to be actively elongating [38]. Therefore, at least a fraction (probably the majority) of transcripts with the highest BoE in S3 Fig were arising from promoters with elongating polymerase type II.

The above trivial correlations with the components of RNA polymerase II complex might be masking more interesting, but subtler, effects that were more likely to be causative. Therefore, we removed Pol II and TAF1 from the second tree model and from all GLMs. We stress that it was previously found that the correlation between the size of promoter architecture and BoE persisted after Pol II sites were disregarded [1]. (If Pol II and TAF1 were retained in the core R GLM, they produced coefficients of 0.675 and 0.3 and were ranked as the first and the third most significant terms.)

Interestingly, the second tree model (Fig 3) had a different regulatory logic to the first one. In the first model (S3 Fig), several types of binding sites *cumulatively* led to high BoE. In the second model, there were *alternative* TFs which separately conferred higher than average BoE, but there was no evidence for their cooperativity. In other words, these TFs were not additive in explaining additional deviance. How can these results be interpreted? Clearly, having either a HEY1, or ELF1, or E2F1, or YY1, or Egr-1, or a *c*-Myc binding site made a transcript’s BoE higher than average. Not having any of these sites made transcript’s BoE as low as not having any Pol II binding sites. (The average BoE of such Pol II-depleted transcripts was lower than 0.1 which corresponded to less than 10% of cell-lines expressing the transcript.)

Interestingly, the pro-housekeeping TFs identified by the second tree model (Fig 3) tended to have a variety of diverse targets and context-dependent roles in many tissue-types. For example, Egr-1 and *c*-Myc integrate many different signalling pathways. Their aberrant expression may be associated with cancer, but low levels of expression are present throughout tissue and cell-types and developmental stages. Egr-1 is a transcriptional regulator of many biological responses in cell types that are widely distributed among the tissues of the body: fibroblasts, endothelial cells, epithelial cells and lymphocytes [39, 40]. The second TF, *c*-Myc modifies expression of up to a third of transcriptome and was proposed to act as a universal amplifier of gene expression [41, 42]. According to the modern view, *c*-Myc, although originally identified as a proto-oncogene, does not have its own unique transcriptional program. Instead, it strongly amplifies transcriptional output of genes that are already active, perhaps by prompting paused Pol II to enter the phase of elongation [43]. Crucially, *c*-Myc does not localize to the promoters of silent genes. However, *c*-Myc was identified as a key cooperative binder with a group of pioneer TFs which can recognize and bind silent chromatin and initiate the reprogramming of somatic cells to pluripotency [44]. Moreover, a systematic search for pioneer TFs which can open chromatin and activate transcription [45] identified several TFs which in our data correlate positively with the high BoE. These included Nrf1, E2F1, and Elf1, while c-Myc was identified as a key settler TF. All these facts suggest a causative mechanism for *c*-Myc.

Several TFs identified as model terms with negative coefficients to BoE were known transcription repressors, lineage-specific differentiation factors, or TFs involved in inducible rather than housekeeping expression (Table 2). This is straightforward to interpret in causative terms, as lineage-specific or inducible expression patterns disagree with housekeeping expression. For example, SUZ12 is a member of the polycomb repressive complex that functions in the heterochromatin-mediated repression of transcription [46]. TAL1 plays a role in the differentiation of the hemopoietic lineage [47]. ZNF274 is another transcriptional repressor [48]. It recruits histone methyltransferase SETDB1 to the 3’-ends of zinc finger proteins, thus effectively silencing multiple TFs [49]. Another TF negatively impacting BoE, NRSF is a well-known transcriptional repressor which restricts the expression of sodium channel genes to neurons [50, 51]. Yet another negative regulator of BoE, *c*-Fos, is a proto-oncogene whose expression tends to be inducible and temporarily restricted [52, 53]. Finally, MEF2A, or a myocyte enhancer factor 2A, activates the expression of muscle-specific genes and plays a role in muscle development and neuronal differentiation [54].

We note that some concern was expressed in the literature that modENCODE [55] ChIP-seq peaks may coincide with spurious Phantom peaks [54]. Such phantom peaks might be due to non-specific interactions of ChIP-seq antibodies with the regions of active transcription (where the chromatin has open structure and the presence of activated but unstructured proteins might randomly attract other proteins). Here, we do not comment on the modENCODE data. However, we did previously discuss and reject a conceptually related model of spurious TF binding termed a “sticky” model [1]. The findings presented here add further weight to the evidence against the “sticky” model, because the binding patterns of TFs are clearly non-random and their impact on BoE is easily interpretable.

The total number of TFs in the human genome was proposed to be as high as 1,391 [56]. This would suggest that the ChIP-seq screen of ENCODE investigated perhaps as little as 10% of the full repertoire of human TFs. Nevertheless, it should be correct to extrapolate the trends detected in this subset. Could the intercepts of the linear models be used to infer any information about the “missing” TFs? The intercepts of different GLMs were strongly converging (with the mean of -2, or -2.4 for a model with interactions). This corresponds to the BoE of 12–9% (the inverse logit between 0.12 and 0.09). In other words, transcripts with empty promoter architectures were on average expressed in approximately one tenth of the samples. This basal expression level is probably the footprint of the TFs missing from ENCODE.

The regularization and shrinkage performed by lasso, ridge and elastic net ought to eliminate TFs with little impact from the models of BoE. Did they lead to the elimination of many TFs from the models? The answer is mostly negative. See Table 5 for the summary of different models. The table contains the numbers of variables included in the models. At most 22% of TFs were irrelevant in the elastic net model. This is strong evidence for the non-redundant role of individual TFs. In other words, the regulation of BoE could not have been reduced to the actions of just a few TFs. Moreover regularization did not improve model fit (Table 6).

**Table 5:** The summary of different models.

Model type		Implementation	Model coefficients (*θ*_*1-N*_)					
			Fraction of coefficients which were…			Min	Max	Span
			Positive	Negative	Zero			
Linear-response	Regularized GLM	*Glmnet* lasso	0.53	0.37	0.1	-1.41	0.43	1.84
		*Glmnet* ridge	0.57	0.4	0.03	-1.47	0.39	1.86
		*Glmnet* elastic net	0.46	0.32	0.22	-0.57	0.41	0.98
	GLM	Core R	0.56	0.41	0.04	-9.26	0.44	9.70
		Bayesian	0.47	0.53	0	-8.1	13.5	21.6
Non-linear-response (cluster II)	Regularized GLM	*Glmnet* lasso	0.25	0.02	0.72	-0.72	0.71	1.43
		*Glmnet* ridge	0.80	0.188	0.014	-0.54	1.03	1.57
		*Glmnet* elastic net	0.31	0.051	0.638	-0.89	0.83	1.72
	GLM	Core R	0.59	0.399	0.014	-7.0	1.5	8.45
		Bayesian	0.55	0.42	0.03	-13.7	9.9	23.6

**Table 6:** Pairwise TF interactions improve model fit but regularization does not. The fact that the statistical model with pairwise TF interactions had the lowest fit proves the significance of synergies and antagonisms between TFs.

Model (linear response)		Cross-validation error	Residual deviance
Quasibinomial GLM		0.0487	7736343
Quasibinomial GLM with pairwise interactions		0.0529	6518074
Regularized models	Lasso	0.05	7783521
	Ridge	0.05	7789755
	Elastic net	0.05	7784786
Random forest (148 variables)		0.066	n/a

NOTE: the cross-validation error was calculated using the mean squared error and 10-folds. Residual deviance is a goodness-of-fit statistics which can be calculated for models fit by maximum likelihood. Null deviance equalled 11544000.

In fact, neither the interactions, nor regularization, nor a fully non-linear model built with random forest improved predictive performance over the core R GLM as measured by cross-validation (Table 6). In the case of regularization, the failure to improve predictions was not entirely unexpected as the goal of regularization was variable selection and coefficient shrinkage. However, that a fully non-linear modelling technique, the random forest, did not improve predictive performance was significant, suggesting that most signal encoded in the data was already recovered by the linear models.

**Fig 7:**
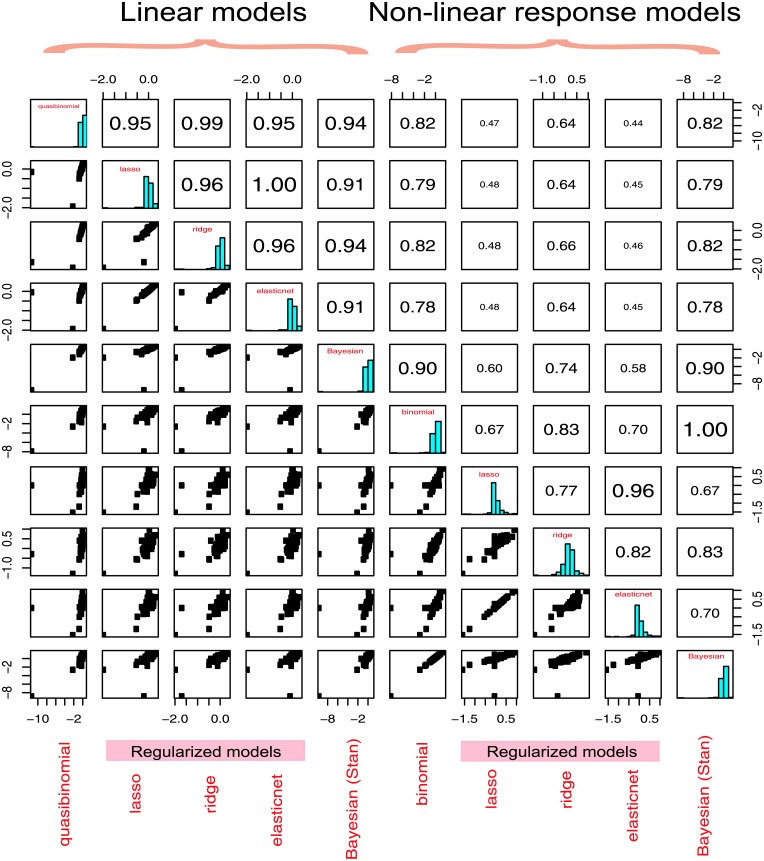
The comparison of models. The agreement between the models is expressed in the correlation between the vectors of coefficients produced by different models. This figure shows the matrix of scatterplots for pairwise comparisons between: the binomial GLM, lasso, ridge, the elastic net, the Bayesian model, together with the three cluster (I, II and III) of the non-linear-response model. The panel above the diagonal shows pairwise correlation coefficients (Spearman correlation) between the vectors of regression coefficients *β*_*i*_. On the diagonal, a histogram of coefficients from each of the models is displayed. The classical GLMs are very similar to each other (with Spearman’s *rho* between 1 and 0.97). The Bayesian model is slightly more diverged (*rhos* between 0.92 and 0.93).

Were the models produced by non-regularized and regularized GLMs similar? Their coefficients were highly correlated (Fig 7). As the ratio of the number of transcripts to the number of explanatory variables was high (116) over-fitting was probably less of a concern resulting in a limited correction by the shrinkage of coefficients. (Typically over-fitting manifests itself in very large values of regression coefficients). However, some shrinkage was introduced by the regularization methods: this was evident by the lower variances and smaller spans of the resulting vectors of coefficients (Table 5, the average non-regularized span was 15.84, versus the value of only 1.57 for regularized models, Wilcoxon *P*-value = 0.0095). Careful examination of the vectors of coefficients suggested a few further generalizations. Between 46–57% of TFs were pro-housekeeping in the linear-response models. For the non-linear-response models, this fraction varied widely, with 54–59% for non-regularized models, and falling as low as 31% and 25% for the elastic net and lasso. The last model was an outlier with most coefficients shrunk to zero by regularization. For most models, the fraction of TFs with positive coefficients was higher than those with negative (the average difference was 20%, paired t-test *P*-value = 0.0048). The last observation contributed to the explanation of the general correlation between the size of the architecture of the promoter and BoE. The net effect of the increasing size of the architecture of the promoter was in increasing BoE.

By adding Bayesian GLMs as an alternative linear and an alternative non-linear-response model, we further ensured that the findings presented were robust in respect to the statistical methodology employed. The classical linear models were more similar to each other than to the Bayesian model, however, the congruence with the Bayesian model was still high (Fig 7). For example, the mean correlation between classical linear models was *rho* = 0.97, SD = 0.02, N = 6, while the mean correlation with the point estimates from the linear Bayesian model was *rho* = 0.93, SD = 0.02, N = 4 (Wilcoxon test between the two groups of comparisons *P*-value = 0.0095).

In summary, we constructed several explanatory statistical models of the breadth of expression in human. The models were largely in agreement in identifying a group of pro-housekeeping transcription factors (the majority of ENCODE TFs), and a small group of repressors which disagreed with housekeeping expression. Moreover, the theoretical knowledge of transcriptional regulation suggested these associations were likely to be causative. The results presented here should impact on our understanding of the regulation of gene expression.

## Methods

### Statistical tests

Contingency tables: the *P*-value for rejecting the null hypothesis of independence of rows and columns in contingency tables was calculated by Monte Carlo simulation (based on 2000 replicates). The *P*-value was calculated by core R function *fisher*.*test()*, which implements a C version of the FORTRAN subroutine FEXACT [57, 58].

The comparison of means of two samples: in most cases, the variables compared were not normally distributed and the Wilcoxon test was used. The author refers to the two-sample Wilcoxon rank sum test with continuity correction, as implemented in the function *wilcox*.*test()* of core R [59]. This function computes exact *P*-value through permutations (but only if there are no ties). In other cases a normal approximation is used.

### ENCODE data, F5 data, and data integration

To define promoter architectures, we used merged ChIP-seq peaks [60, 61] from the ENCODE consortium [62]. For gene expression, we used F5’s [7] work package 4 expression tables [63]. ENCODE and F5 datasets were pre-processed and integrated as described in detail previously [1].

### BoE

In the simplest approach, BoE was modelled as a random variable which varied from zero through to one. This simple representation was used in tree models. However, for GLMs BoE was re-interpreted as the number of successes (expression switched “on”) in the total number of attempts. Each attempt was one tissue or cell-line. Thus, for the GLMs, the response variable was re-coded as a two-row matrix. One row was the number of successes (samples with expression “on”). The second row was the total number of attempts. The total number of samples equalled 1660, *i*.*e*. the number of libraries in the F5 dataset.

### Correlogram

We computed the correlogram by performing a hierarchical clustering of the Spearman’s correlation coefficients with a complete linkage method aiming at finding similar clusters. We then plotted the coefficients in the lower triangle of the correlation matrix. Colour-coding was used to highlight the significant values of Spearman’s *rhos*. *P*-values were obtained from the evaluation of correlations using their random expectation.

### The tree models

The tree model algorithm is also known as binary recursive partitioning. The tree algorithm splits the dataset in consecutive steps at each step choosing the variable which maximally contributed to explaining the deviance of the response variable. The splitting continues until the data are too sparse to proceed further (which means fewer than six observations left to partition). The algorithm was implemented in the R function *tree* from the package under the same name [21, 64]. The models are shown in Fig 3 and S3 Fig.

### Linear-response GLM: The core R

GLMs are a family of models which generalize linear regression to response variables for which errors are not normally distributed. To specify a model one specifies the family of distributions from which errors derive and a link function. The link function can be non-linear and describes the relation between the mean of and the linear combination of the predictive variables in the model [21]. BoE was conveniently modeled as a proportion [21]: the number of samples a gene was expressed in over the number of samples. This suggested a binomial structure of errors [21]. Accordingly, we chose GLMs of the quasi-binomial family (with the *logit* canonical link function). The logistic link function has a “squashing” effect, transforming real numbers into the interval [0, 1]. Quasi-likelihood was used to compensate for over-dispersion of the linear model (the residual deviance of 1,980,000 at 30,792 degrees of freedom). The GLMs produced vectors of correlation coefficients *θ*_*1-N*_ (for *N* TFs which were the inputs for the model) plus the estimate for the intercept (*θ*_*0*_). Model parameters were by default returned on the scale defined by the reverse of the link function. These values could be transformed into the scale of model inputs by a reverse logit transformation. Standardized coefficients were the result of variant analyses where variances of independent and dependent variables were standardized to one. As all independent variables were on the same scale (TF counts), standardization was not essential. Unless otherwise stated, interaction terms were not included in models.

### Linear-response GLMs with regularization (lasso, ridge, and the elastic net)

Lasso [30] and ridge [65] are likelihood penalized GLMs implemented in the R package *glmnet* [66]. The key feature of *glmnet* is regularization which facilitates variable selection and shrinkage among the groups of correlated explanatory variables. This makes *glmnet* the method of choice for genomics applications (where the number of features tends to be high and the number of observations may be limited). The authors of the package use the metaphor of the fishing net catching “big fish”. The net ensures that only significant variables are selected to have non-zero coefficients in the final model [66]. Depending on the value of the parameter alpha, *glmnet* will favor either the lasso (alpha = 1) or the ridge (alpha = 0). The elastic net (alpha = 0.5) is a compromise between lasso and ridge. The ridge penalty tends to shrink the coefficients of correlated variables so that they become more similar. In contrast, the lasso tends to promote one of the variables in a correlated group and discard the others. Both lasso and ridge were invoked through the function *glmnet()*. Lambda is the parameter of regularization controlling the penalty imposed on maximum likelihood. The *glmnet* algorithm constructs a sequence of lambda values and plots the correlation coefficients of the model along this sequence (S4 Fig). It is recommended that correlation coefficients at *lambda*.*min* are picked for the final model.

### *Glmnet* cross-validation identifies lambda values at which the prediction error is at the minimum

*Glmnet* automatically builds a predictive model and performs leave-one-out cross-validation. Although, a prediction was not our goal, cross-validation is a crucial means of the verification of the fit of explanatory models [12]. The cross-validation reports *lambda*.*min*–the value of lambda which minimizes the cross-validated mean squared error (S5 Fig). For example, in our linear GLMs, the values of *lambda*.*min* were 0.0005 for the lasso, 0.00046 for the ridge, and 0.0009 for the elastic net. These values of lambda were used to report the *glmnet* correlation coefficients of individual TFs.

### Bayesian GLMs with R and Stan

Stan is a powerful and flexible statistical system and programming language for Bayesian statistical modelling [67, 68]. Stan performs fast Markov-chain Monte Carlo (MCMC) calculations of posterior distributions with a No-U-Turn sampler. *Rstan* is a general R interface to Stan; *Rstanarm* is a more specialized R interface for GLMs.

For our Bayesian model, we used weak non-informative Gaussian priors ***Ɲ***(0,10) for both the regression coefficients (*θ*_*1–148*_) and the intercept (*θ*). The model was defined with an identical formula to the one used for *glm()* and *glmnet()*. The MCMC sampler was invoked using *Rstanarm’s* function *stan_glm()*. Four MCMC chains were run for 1,000 iterations (split evenly between warm-up and sampling).

The convergence between the chains was estimated using $\hat{\text{R}}$. $\hat{\text{R}}$ is the so-called potential scale reduction factor for MCMC chains. It is interpreted as a potential further improvement in convergence, expected if the chains were allowed to run indefinitely. At convergence, $\hat{\text{R}}$ approaches one.

### *K*-means clustering

The *k*-means implementation of the core R was used, namely the function *kmeans()*. The option of the Forgy algorithm was specified. The clusters were defined in two dimensions. These clusters were used to construct a non-linear-response model with multinomial regression that was fitted with the *glmnet* package using the lasso penalty (alpha = 1).

### The details of the non-linear-response modelling

The core R and the *Rstanarm* implementations of GLMs do not implement multinomial regression. Therefore, the response variable of multinomial regression had to be recoded as a binary variable (cluster II versus the other two clusters).

### Random forest

A 148-variable random forest was built using R package *randomForest*. Cross-validation was performed using 10-folds.

## Supporting information

S1 Fig Correlogram. This supplementary figure shows the correlogram of TFs color-coded by the strength and significance of their Spearman’s pairwise correlation coefficients.
(EPS)

S2 Fig Random expectation. This supplementary figure contains a plot visualizing the random expectation of TF-correlations compared with the observed distribution. The determined thresholds of statistical significance are also visualized.
(EPS)

S3 Fig A tree model of BoE. RNA Polymerase II, TAF1 and S2-phosphorylated Pol II sites were the most significant sources of variability.
(EPS)

S4 Fig Regularization paths. The regularization paths of: lasso, ridge, and the elastic net for the linear-response (A-C) and non-linear-response models (D-F) models are visualized in this supplementary figure.
(EPS)

S5 Fig *Glmnet* cross-validation. *Glmnet* performs leave-one-out cross-validation after the data-set is grouped into ten folds (random partitions). The value of *lambda*.*min* reported by cross-validation is the value in the lambda sequence (visualized along the horizontal axis) which minimizes the cross-validated binomial deviance (vertical axis) and was used to report the values of the coefficients of the model. Panel *A* shows the cross-validation results for the lasso, panel *B* for the ridge, and panel *C* for the elastic net version of the GLM regression. Panel *D* shows cross-validation for the multinomial lasso of the non-linear-response model.
(EPS)

S1 Dataset RefSeq transcripts. (TXT)

S2 Dataset TF-transcripts matrix. Note this dataset contains redundant RefSeqs. Please, contact the corresponding author for details on post-processing and R scripts.
(TXT)

S1 Table Correlated TFs. All pairs of correlated TFs are given in this supplementary table.
(TXT)

S2 Table The linear response GLM: Core R with TF interactions. Model terms correspond to ENCODE TFs cut off at the significance level of 0.05 (linear model, quasibinomial likelihood, core R GLM). The table also lists pairwise TF interactions. The intercept is also shown on the logit scale.
(TXT)

S3 Table The comparison of coefficients of GLMs. Correlation coefficients of different models were compared in this supplementary table.
(TXT)

S4 Table Variable importance of the random forest model. (TXT)
