# Correction: Modelling of the breadth of expression from promoter architectures identifies pro-housekeeping transcription factors

**DOI:** 10.1371/journal.pone.0227416

**Published:** 2019-12-30

**Authors:** Lukasz Huminiecki

There are errors in the Funding statement. The correct Funding statement is as follows: LH was funded to perform this work by the National Science Centre, Poland, grant POLONEZ 2 (grant number 2016/21/P/NZ2/03926). This project has received funding from the European Union’s Horizon 2020 research and innovation programme under the Marie-Sklodowska-Curie grant agreement No.665778. The funders had no role in study design, data collection and analysis, decision to publish, or preparation of the manuscript.
